# Genome-wide identification and expression analysis of the 2OG-Fe(II) oxygenase gene family in upland cotton (*Gossypium hirsutum L.*)

**DOI:** 10.1007/s12298-021-01065-4

**Published:** 2021-09-25

**Authors:** Fenglei Sun, Quanjia Chen, Qin Chen, Menghui Jiang, Qingtao Zeng, Yanying Qu

**Affiliations:** 1 Engineering Research Centre of Cotton, Ministry of Education, College of Agriculture, Xinjiang Agricultural University, 311 Nongda East Road, Urumqi, 830052 China; 2 Agricultural Science Institute, Seventh Agricultural Division of Xinjiang Production and Construction Corps, Kuitun, 833200 China

**Keywords:** 2OG-Fe(II) oxygenase, Synteny, Genome-wide analysis, Drought stress, Cotton, Leaf curling

## Abstract

The 2OG-Fe(II) oxygenase (RF) family of enzyme proteins can affect bulliform cells and cause leaf curling. However, there are few studies related to this family in cotton, and there has been no systematic analysis of RF genes. Here, we determined 25 RF genes in the complete genome sequence of upland cotton (*Gossypium hirsutum L.*) and 11 RF genes in the complete genome sequence of *Arabidopsis thaliana*. Cotton RF proteins can be divided into three categories. Whole genome/fragment and scattered replication events played an important role in the expansion of the RF gene family. qRT-PCR analysis results showed that RF genes respond to drought stress Pairwise comparison results showed that the expression of RF genes in Shi yuan 321 was higher than that in Kui 85–174. Overall, genome-wide identification approach was used to further analyze the related functions of the RF gene family, which may include the response to drought stress, in cotton.

**Supplementary Information:**

The online version contains supplementary material available at 10.1007/s12298-021-01065-4.

## Introduction

Drought stress affects crop growth and physiological metabolism and thereby reduces crop yield (Nagy et al. 2013; Liu et al. 2015). Therefore, in the process of evolution, crops have developed defense mechanisms and metabolites to deal with biotic and abiotic stresses (Zhang et al. 2019). Crops adjust their transpiration rate and light-receiving area through leaf curling to improve tolerance to drought stress (Fang et al. 2011). As water transport is affected, the growth of blister cells is affected, causing the leaves to curl (Alvarez et al. 2008; Wu et al., 2012). The 2OG-Fe(II) oxygenase gene family affects water transport by affecting the synthesis of secondary cell walls. The reduction in water transport affects bullous cells, making them abnormal (Fang et al. 2011). Analyses of some leaf development mutants of Arabidopsis and maize have shown that some mutations used for leaf curling are related to the development of leaves along the front and rear axes (Fang et al. 2011). The development of leaves along the front and rear axes may be controlled by transcription factors and small RNAs (Moon and Hake 2010). Some of these genes belong to the HD-ZIP III family of genes (such as PHABULOSA (PHB), PHAVOLUTA (PHV), REVOLUTE (REV) and ROLLED1 (RLD1)), which determine the development of adaxial cells in leaves (McConnell et al. 2001; Otsuga et al. 2001; Nelson et al. 2002). In contrast, the MWP1 and YAB families (such as the YAB2 and YAB3 genes) determine the development of abaxial cells (Candela et al. 2008; Eshed et al. 2004). Additionally, the growth and development of vesicular cells or osmotic pressure are also important factors that affect the formation of leaf curls (Alvarez et al. 2008). At present, few studies have identified the role of 2OG-Fe(II) oxygenase genes in response to drought stress. The identification of relevant drought resistance genes is essential to provide good candidate genes for drought resistance breeding in upland cotton. Therefore, the study of 2OG-Fe(II) oxygenase genes is of great significance.

The development of sequencing technology has helped sequencing of many genomes including the genome of upland cotton. The genome-wide analysis of different gene families provides us with important information on the response mechanism of cotton drought stress and other abiotic stresses, and provides an important theoretical basis for future research (Yang et al. 2019).

The 2OG-Fe(II) oxygenase (RF) gene family has been studied in rice, and is mainly found to be involved in leaf curling and regulating photosynthesis (Fang et al.^2011^). However, less information is available on the RF gene family in cotton, and few studies have investigated RF genes and their role in drought tolerance. We conducted the first genome-wide identification and analysis of the RF gene family of upland cotton. In our study, 25 RF genes were identified from upland cotton. GhRF gene expression data from six different tissues were extracted from the cotton database. Furthermore, the expression of 25 RF genes in different tissues was studied and the response of 25 GhRF genes to drought stress was analyzed. This study identified and classified the cotton RF gene family through a systematic evolutionary analysis, and provided a theoretical basis for future research on the cotton RF gene family.

## Materials and methods

### Sequence identification of 2OG-Fe(II) oxygenase proteins

The serial group data and annotation data of upland cotton were obtained from Cotton FGD (https://cottonfgd.org/) (Zhu et al.^2017^). Arabidopsis genome data were obtained from the Phytozome v12 database (https://phytozome.jgi.doe.gov/pz/portal.html).

The hidden Markov model (HMM) (PF13532) in the Pfam database was downloaded with “2OG-Fe(II) oxygenase proteins” as the keyword (https://pfam.xfam.org/) (El-Gebali et al. 2019), and the sequences containing “2OG-Fe(II) oxygenase proteins” were checked through HMMER3.0 (e value 10^–5^). Members of the 2OG-Fe(II) oxygenase gene family were identified. Through the Pfam database and NCBI-CDD, the conserved domains of 2OG-Fe(II) oxygenase proteins were further determined, and redundant sequences were deleted. The ProtParam and CELLO RESULTS (https://cello.life.nctu.edu.tw/) online tools (https://web.expasy.org/protparam/) were used to calculate the physical and chemical properties and subcellular localization of 2OG-Fe(II) (Yu et al., 2006). These 2OG-Fe(II) candidate genes (RF) were named according to their chromosomal position (bp) from the At subgenomic chromosome to the Dt subgenomic chromosome.

### Motif analyses, gene structure and phylogeny

The conserved domains of RF proteins were analyzed by MEME (https://meme.sdsc.edu/meme/cgi-bin/meme.cgi), and the CDS structures were analyzed through TBtools visualization (Chen et al. 2020). The RF protein sequences of cotton, *Oryza sativa* and *Arabidopsis* species were analyzed by MEGA 7.0, and a phylogenetic tree was constructed. The neighbor joining (NJ) method and bootstrap analysis with 1000 replicates were used for data comparison to construct the phylogenetic tree.

### Chromosomal position and collinearity analysis

The specific staining position of the RF gene was obtained by searching the Phytozome database and Cotton FGD using the default settings. The RF genes were present on different chromosomes of cotton, from the At subgenomic chromosome to the Dt subgenomic chromosome, and finally a chromosome distribution map was drawn by TBtools software. RF protein sequences of upland cotton and *Arabidopsis thaliana* genomes were analyzed by homology, and the gene replication events between upland cotton and *Arabidopsis thaliana* were analyzed and visualized by TBtools. TBtools software was used to calculate the synonymous substitution rate (ks), nonsynonymous substitution rate (ka) and ka/ks between homologous gene pairs and to analyze the selection pressure during the evolution of the GhRF gene family.

### Transcriptome of RF genes in cotton

The RF gene FPKM (fragments per kilobase per million reads) values were downloaded through the Cotton FGD (https://cottonfgd.org/) database to analyze the expression values of the cotton RF genes. Finally, a heat map of RF gene expression in cotton was drawn by TBtools.

### Plant material, drought stress treatment and quantitative real-time PCR (qRT-PCR) analysis

Shi yuan 321 and Kui 85–174 were provided by the genetic breeding laboratory of Xinjiang Agricultural University. According to previous laboratory experiments, Shi yuan 321 is a drought-resistant cotton variety, while Kui 85–174 is a drought-sensitive variety. We used 0.5% sodium hypochlorite (NaClO) and sterile distilled water to sterilize upland cotton seeds (Shi yuan 321 and Kui 85–174) (Wang et al. 2019). Seeds were sown in high-temperature sterilized soil and grown to the three-true-leaf stage under a 16-h light/8-h dark cycle (Chen et al. 2020). Soil drought stress treatment was carried out, and cotton roots, stems, and leaves were collected at 0, 1, 3, 6, 12, and 24 h after the stress treatment. The collected tissue samples were stored in an ultralow temperature refrigerator at -80 °C. Total RNA was extracted with an RNA kit (TIANGEN Total RNA Extraction Kit, TIANGEN Biochemical Technology (Beijing) Co., Ltd.), and first strand cDNA was synthesized by reverse transcription (5X All-In-One RT MasterMix, abm Biotechnology (Canada) Co., Ltd.). The PCR products were verified by 2% agarose gel electrophoresis (electrophoresis detection of cDNA of 25 cotton RF genes, and the PCR conditions were as follows: pre-denaturation 25 °C for 10 min, denaturation at 42 °C for 15 min, extension at 85 °C for 5 min). qRT-PCR was performed in an ABI Prism7500, and each sample was repeated three times. The *UBQ7* gene was used as the endogenous standard control. The 2^−∆∆Ct^ method was used to analyze the expression of the cotton RF genes.

## Results

### Identification of RF genes in upland cotton and Arabidopsis

The same genes and redundant sequences were deleted, a total of 25 RF genes were finally identified. Through the Pfam and NBI-CDD databases, we further analyzed 25 possible RF proteins and confirmed the presence of “RF protein” domains. Finally, there were 25 RF genes in cotton, 11 RF genes in *Arabidopsis thaliana* and 11 RF genes in *Oryza sativa*. The isoelectric points and molecular weights ranged between 4.99 to 9.43 and 27,347.96 to 73,593.02 in cotton, respectively. The range of amino acids was between 239 and 682 (aa), but the length of the genes was between 720 and 2049 bp. The results of protein pre subcellular localization analysis showed that 17 proteins were located in the cytoplasm, and the rest were located in extracellular tissues (four) and periplasm (four) (Table S1).

### Phylogenetic classification, gene structure and motif identification analyses of cotton RF genes

**Fig. 1:**
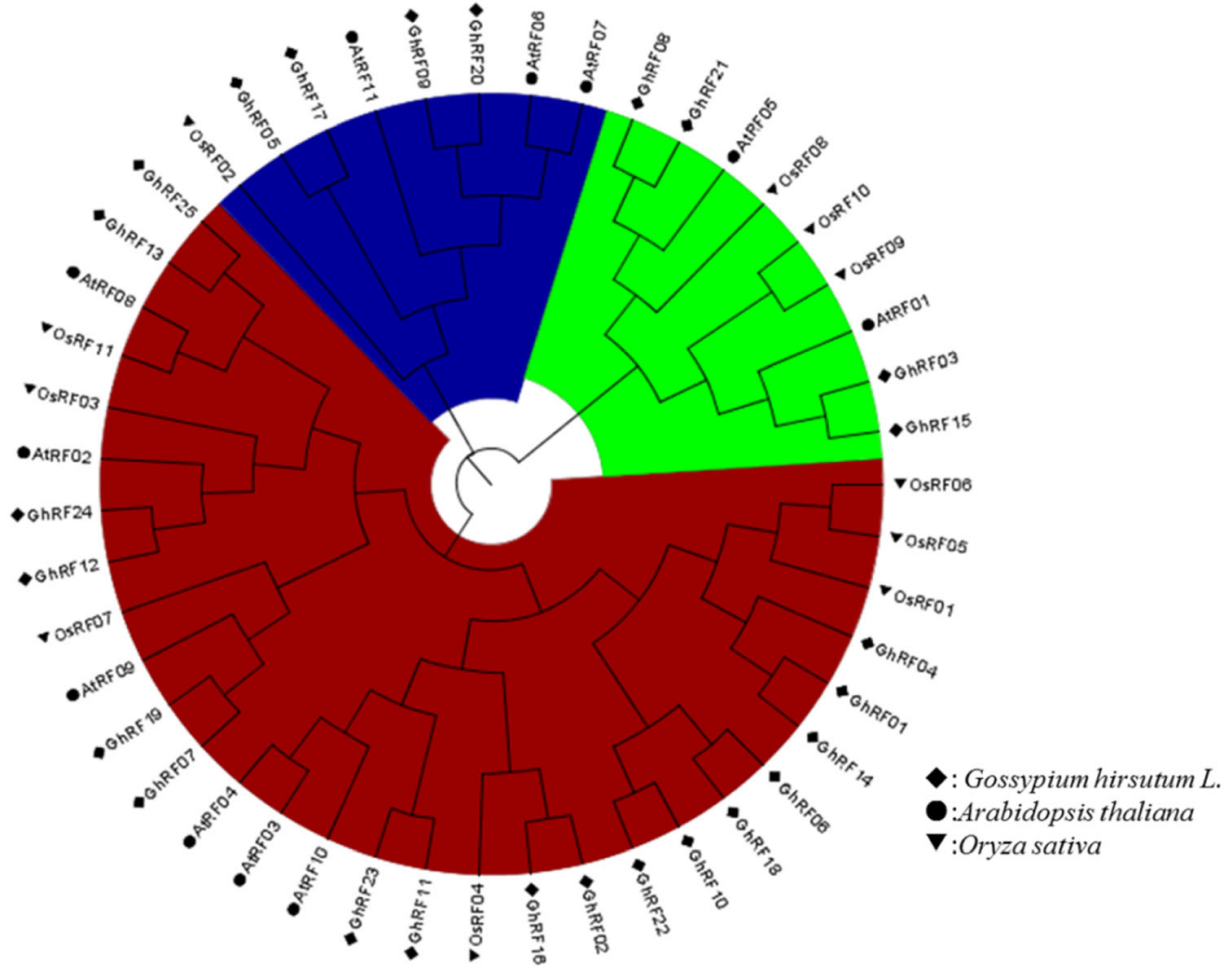
Phylogenetic relationships of cotton RF proteins

**Fig. 2:**
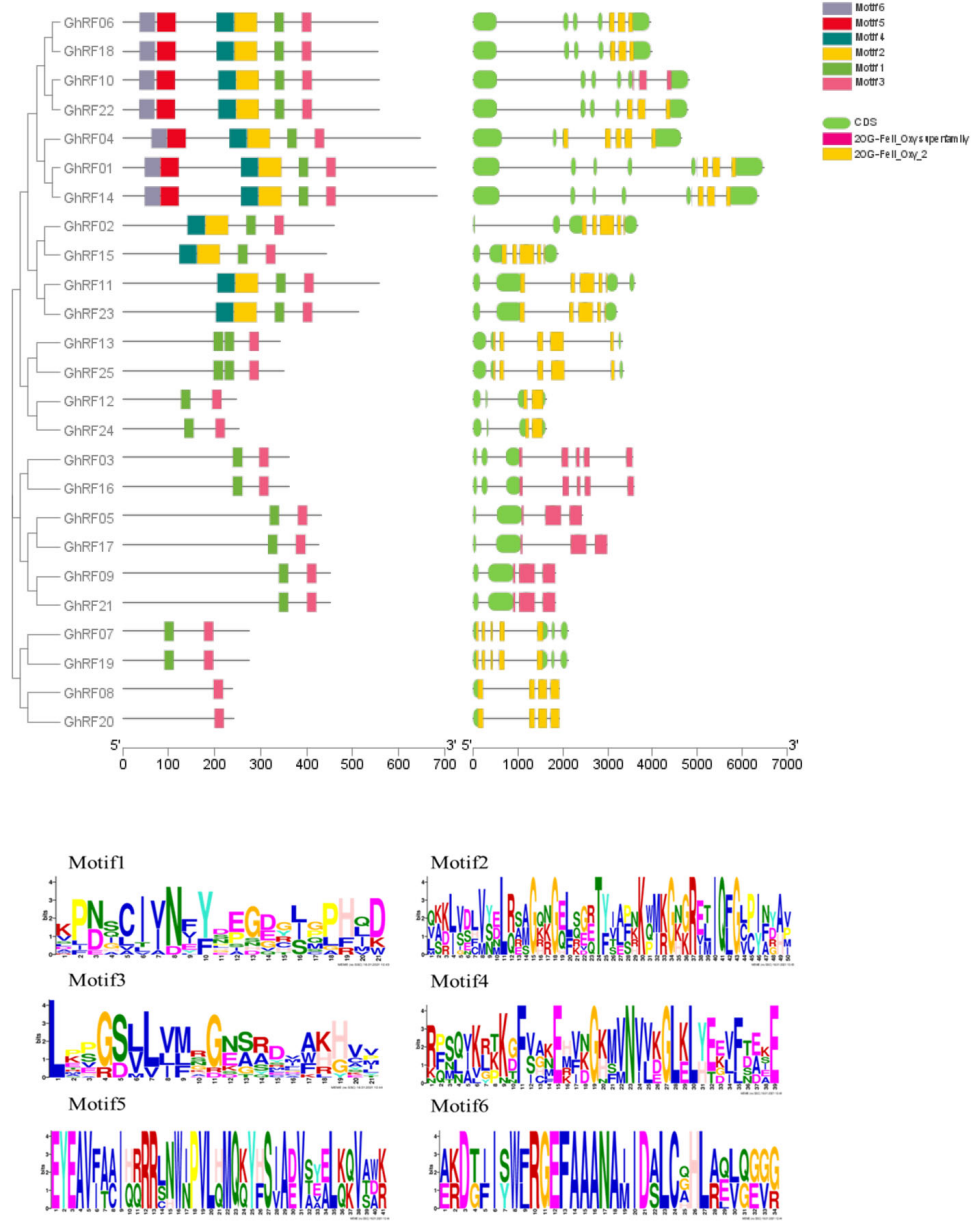
Sequence conservation analysis of RF proteins. **a** Alignment of the conserved domains of RF proteins and genomic exon–intron structures in upland cotton (*Gossypium hirsutum L.*). **b** Six conserved motifs

To identify the evolutionary significance of the RF protein domain in cotton, we conducted a phylogenetic analysis based on the conserved protein domains between upland cotton and Arabidopsis. A large number of branches had highly conserved domains, which indicates that these homologous proteins have similar functions (Fig. 1). According to the results of the phylogenetic tree, the 47 upland cotton, *Arabidopsis thaliana* and *Oryza sativa* sequences were divided into three categories. The first category included 17 members from upland cotton, six members from *Arabidopsis thaliana* and seven members from *Oryza sativa*; the second category included four members from upland cotton, three members from *Arabidopsis thaliana* and one member from *Oryza sativa*; and the third category included four members from upland cotton, two members from *Arabidopsis thaliana* and three members from *Oryza sativa* (Fig. 1). Using HMME online sequencing to predict the motif of cotton RFs, six different motifs were predicted based on the RF conserved domains of upland cotton (Fig. 2). The frequency of amino acids is represented by the height of letters, and the conserved sequence position is represented by the cumulative height of letters. There were 50 highly conserved identical amino acid residues in the conserved domains of all members (Fig. 2). The predicted motif positions of RF proteins in upland cotton can be divided into three categories according to the phylogenetic tree results. Among them, the conserved domain of group II contains one type of prediction motif, while those of groups I and III contain two types of prediction motifs. For example, the conserved protein domains of *GhRF06*, *GhRF18*, *GhRF10*, *GhRF22*, *GhRF04* and *GhRF01* contain the same motif (Fig. 2). The position of the motifs in the conserved domains of the RF proteins may be decisive for the formation of the relative phenotype.

The analysis of the cotton RF gene structures showed that the distribution of exons varied from 4 to 8, indicating a key change in the evolution of the cotton genome (Fig. 2). Among them, the shortest RF gene in upland cotton was *GhRF08* with a full length of 720 bp, while the longest cotton gene was *GhRF01* with a full length of 2049 bp. The clustering results of RF genes in cotton showed a highly similar gene structure among cotton RF homologous genes. Therefore, the differences in gene structure between the RF gene family and members on the same branch *GhRF06*/*GhRF18*, *GhRF10*/*GhRF22*, *GhRF01*/*GhRF14* and *GhRF13*/*GhRF25* were small, and their gene structures were almost the same.

### Chromosome distribution and collinearity analysis of rf genes in upland cotton

**Fig. 3:**
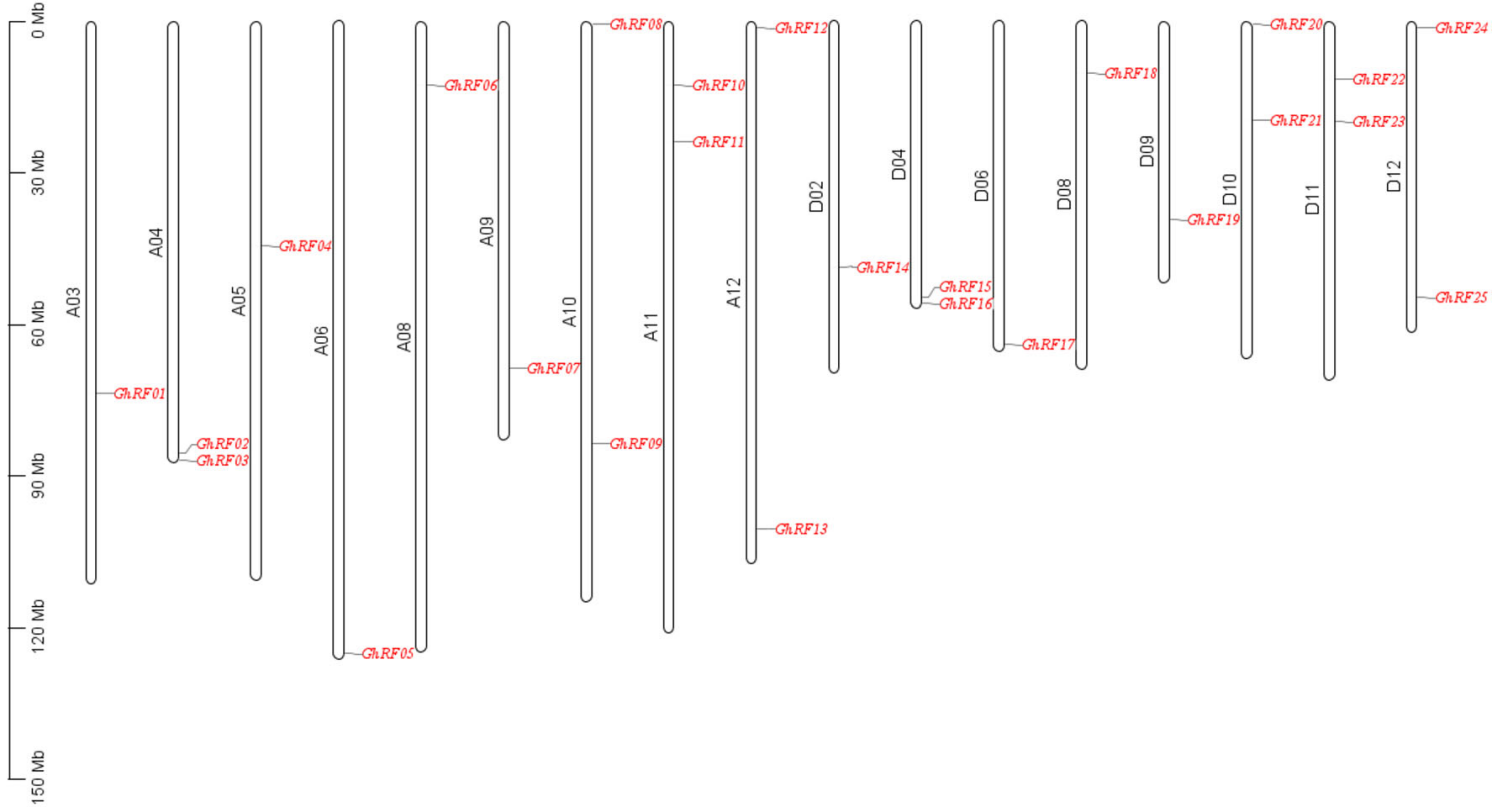
Distribution of 25 RF genes on the chromosomes of upland cotton (*Gossypium hirsutum L.*)

Mapping cotton RF genes onto its chromosomes revealed an uneven distribution (Fig. 3). Table S1 shows the specific locations and sizes of the cotton RF genes on chromosomes. As a result of the analysis, we found that 25 RF genes in *G. hirsutum* were located on 17 chromosomes and were unevenly distributed; among them, nine chromosomes contained only one gene, while eight chromosomes, including AD1-A04, AD1-A10, and AD1-A11, contained two genes (Fig. 3). Except for A03, A05, and D02, the number of genes on the At subgenome chromosome and Dt subgenome chromosome was basically the same. This indicates that some genes may be lost during evolution or that incomplete genome sequencing may cause the number of recognized genes to be less than the actual number of genes. For example, GhRF04_At, located on chromosome A05, has no corresponding orthologs in the Dt subgenome.

**Fig. 4:**
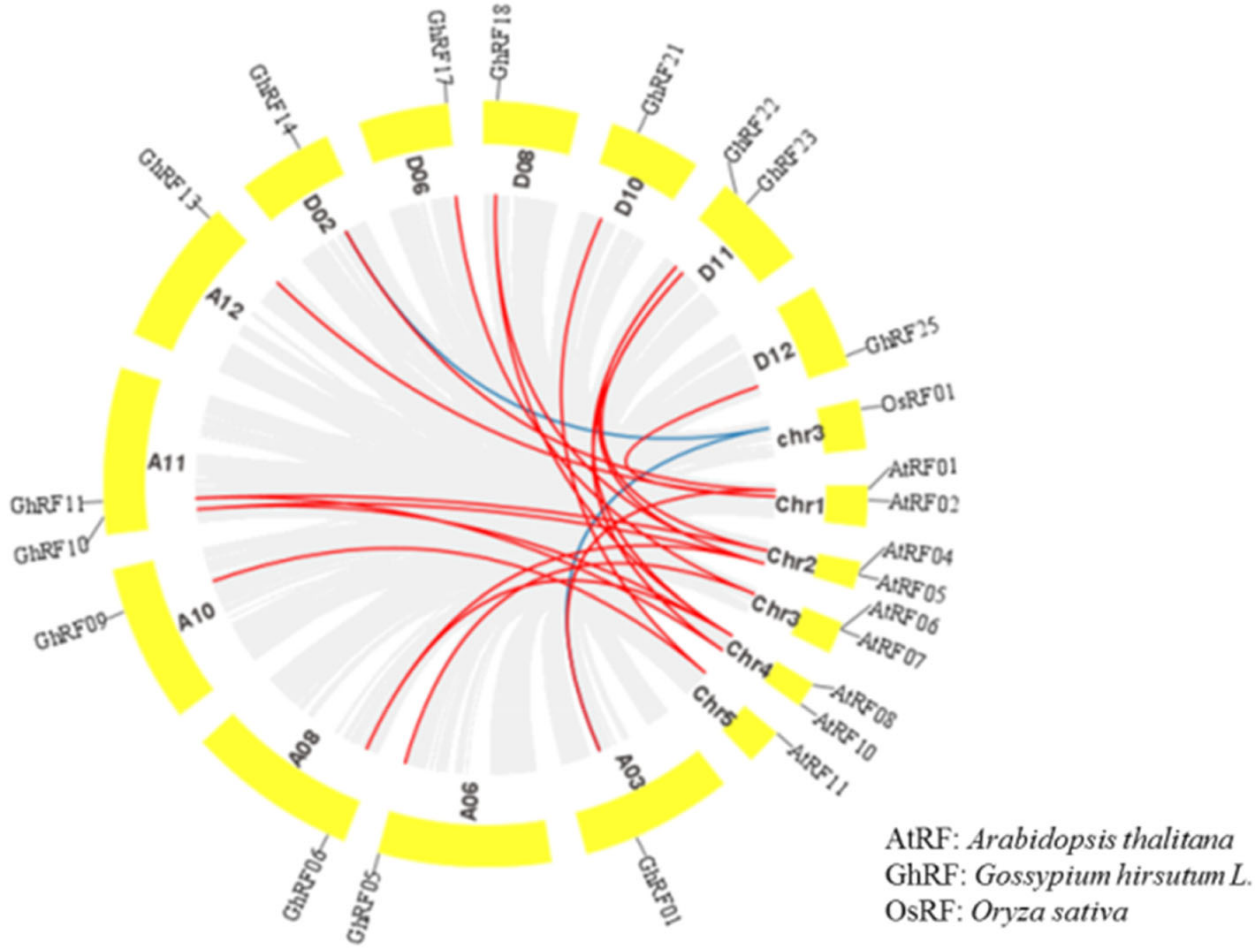
Collinearity analysis and comparison of RF genes in upland cotton (*Gossypium hirsutum L.*) and Arabidopsis

To further understand the collinearity of the RF gene family in upland cotton, we constructed a comparative collinearity map between upland cotton, *Oryza sativa* and *Arabidopsis thaliana*. The RF genes had a greater collinearity relationship between upland cotton and Arabidopsis, but the collinearity relationship between upland cotton and rice was relatively weak. Sixteen RF genes showed a collinearity relationship with those in *Arabidopsis thaliana* (Fig. 4). To further analyze the adaptive evolution of RF genes caused by selective pressure during the evolution process, we calculated the Ka/Ks ratio of each repeated RF gene pair. A Ka/Ks > 1 indicates a positive selection effect, and a Ka/Ks < 1 indicates a purification selection effect. In this study, the Ka/Ks ratio of repeated gene pairs in cotton, rice and Arabidopsis was not greater than 1, which indicates that they underwent purifying selection (Table S2).

### Expression patterns of cotton RF genes in different tissues

**Fig. 5:**
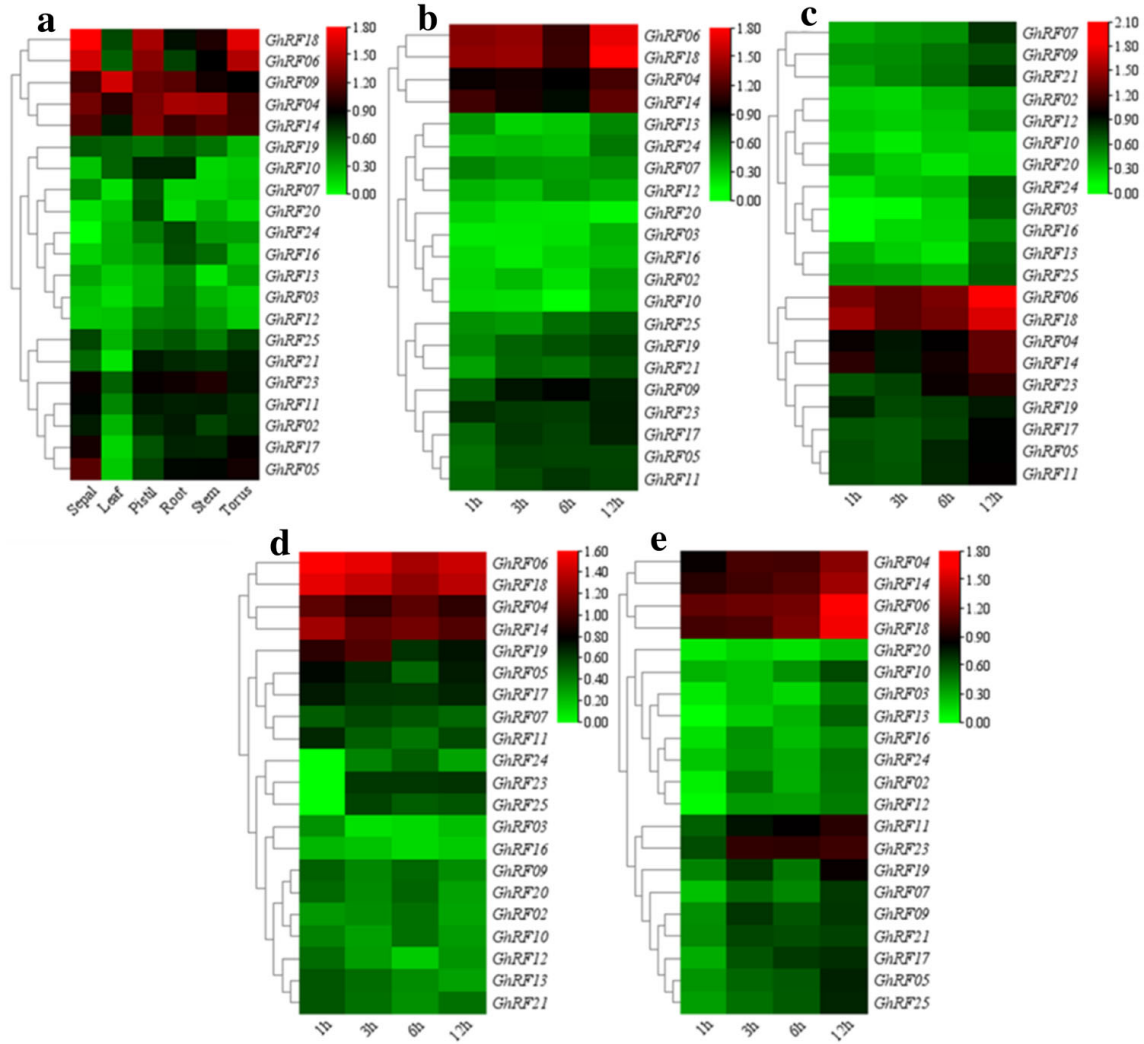
Expression analysis of *GhRF* genes in different tissues under drought stress. **b** Changes in *GhRF* gene expression at different time periods after drought stress. **c** Changes in *GhRF* gene expression at different time periods after salt exposure. **d** Changes in *GhRF* gene expression at different time periods after cold exposure. **e** Changes in *GhRF* gene expression at different time periods after heat exposure

We examined the specific expression patterns of the RF gene in six tissues in upland cotton, including sepals, leaves, pistils, roots, stems, and torus. The results are shown in a heat map, and the expression levels of all genes in the six tissues were different under drought stress (Fig. 5). Some genes, such as *GhRF09*, *GhRF18*, *GhRF04*, *GhRF06* and *GhRF14*, were highly expressed after drought stress induction in these six tissues. These five genes were significantly induced by drought stress.

### Expression analysis of RF genes under drought stress

Gene expression analysis helps to further reveal the related functions of genes. In rice, RF genes are involved in the regulation of plant transpiration and help reduce the transpiration rate under drought stress (Fang et al.^2011^).Except for the expression data of *GhRF01*, *GhRF08*, *GhRF15* and *GhRF22* which we did not examine, the other 21 *GhRF* genes were expressed to varying degrees in the 6 tissues (Fig. 5a). RF genes were induced at different times after drought stress, and some of these genes were expressed at high levels. Five of the RF cotton genes (including *GhRF09*, *GhRF18*, *GhRF04*, *GhRF06* and *GhRF14*) were significantly upregulated (Fig. 5a). Some *GhRF* genes were highly expressed in some tissues after drought stress: *GhRF09* was highly expressed in leaves, pistils, and roots; *GhRF04* and *GhRF14* were highly expressed in sepals, pistils, roots, and stems; and *GhRF06* and *GhRF18* were highly expressed in sepals, pistils, and torus (Fig. 5a). In addition, the *GhRF04*, *GhRF06*, *GhRF14* and *GhRF18* genes all showed a significant trend of upregulated expression within 12 h after drought stress, whereas *GhRF09* also showed a trend of upregulated expression during this time, but this upregulation was not as high as that found with the other four genes (Fig. 5b). The *GhRF04*, *GhRF06*, *GhRF14* and *GhRF18* genes were also significantly upregulated within 12 h after salt stress (Fig. 5c). In response to cold stress and heat stress, the *GhRF04*, *GhRF06*, *GhRF14* and *GhRF18* genes were also significantly upregulated within 12 h (Fig. 5d and Fig. 5e). In contrast, under abiotic stress (drought, salt, cold, and hot), the expression levels of *GhRF04*, *GhRF06*, *GhRF09*, *GhRF14*, and *GhRF18* were increased, which indicates that these genes may play a vital role in the stress response. These genes may be considered candidate genes for further study of cotton stress biology.

## Discussion

In this study, we identified 25 RF genes in cotton and searched for RF genes in *Arabidopsis thaliana*. The physical locations of the RF genes on the chromosomes are shown by the chromosome distribution. The number of RF genes on a given chromosome was small, and the physical location of the chromosome did not affect gene expression. The exon–intron structure also plays a corresponding role in the evolution of diversity (Wang et al. 2015). The rearrangement or fusion of chromosomes of a gene family can cause exon–intron loss and other situations (Xiao et al. 2014). In our study, the number of exons of RF genes in cotton ranged from 4 to 8, with two genes having 8 exons. In Fig. 2, the exon structure of the RF genes in the same branch was very similar. For example, the *GhRF06* and *GhRF18* genes are in the same branch of the phylogenetic tree, and the results showed that they have the same exon–intron structure and number. The difference in gene structure can also reflect the type of gene motif. The results show that different gene structures predict different gene motif types (Fig. 2). Therefore, different RF gene structures may result in different amino acids encoded by the genes and thus different catalytic activities and functions.

Murat et al. (2012) showed that one method of the development of adaptive phenotypes in crops may be genome-wide replication (Murat et al. 2012). At the same time, it may be considered that these genes encoding specific interaction products between biological and nonbiological exogenous factors have retained their corresponding structure and function after replication (Murat et al. 2012; Zu et al. 2019). During the process of plant evolution, the polyploidization mode will cause many repeated genes to appear in the plant genome (Otto et al. 2000). This will cause many changes in gene expression and gene organization in plant tissues (Adams et al. 2007). Therefore, in the process of plant evolution, the polyploidization of plants provides the basic material for this process (Zu et al. 2019). The phylogenetic analysis showed that the homologous RF genes were classified on the same branch (Fig. 1). The RF gene phylogenetic tree and collinearity analysis results showed that the homology, phylogeny and gene structure of the RF genes were basically consistent with the above viewpoints (Figs. 1, 2, 3 and 4). During the process of evolution, the copied genes may undergo corresponding functional differentiation, including functionalization (loss of original function), new functionalization (acquisition of new functions) or subfunctionalization (division of original functions) (Prince and Pickett, 2002; Vandepoele, 2003). To study whether evolutionary selection is related to the difference in RF genes after replication, the nonsynonymous difference level (Ka) and the synonymous difference level (Ka) of 14 homologous pairs were calculated. Based on the Ka/Ks ratio, we can speculate on the selection pressure of replicated genes. In this study, we found that the Ka/Ks values of repeated gene pairs were all less than 1, specifically, the Ka/Ks ratios of 11 gene pairs were less than 0.5, and the Ka/Ks ratios of the remaining 3 gene pairs were between 0.5 and 0.8. (Table S2). Therefore, we speculate that the cotton RF gene family has undergone strong selection pressure for purification, and the functional differences that occur after fragment replication and genome-wide replication are limited. The study by Fang et al. (2011) showed that RF genes affect the normal growth of bullous cells and water transport, which in turn affects the photosynthesis of crops. Moderate leaf curling helps crops absorb light and increases dry matter accumulation, thereby increasing yield (Lang et al. 2004), which shows that RF genes play an important role in regulating plant photosynthesis (Fang et al. 2011). Therefore, the high expression of cotton RF genes helps improve the drought tolerance of cotton.

**Fig. 6:**
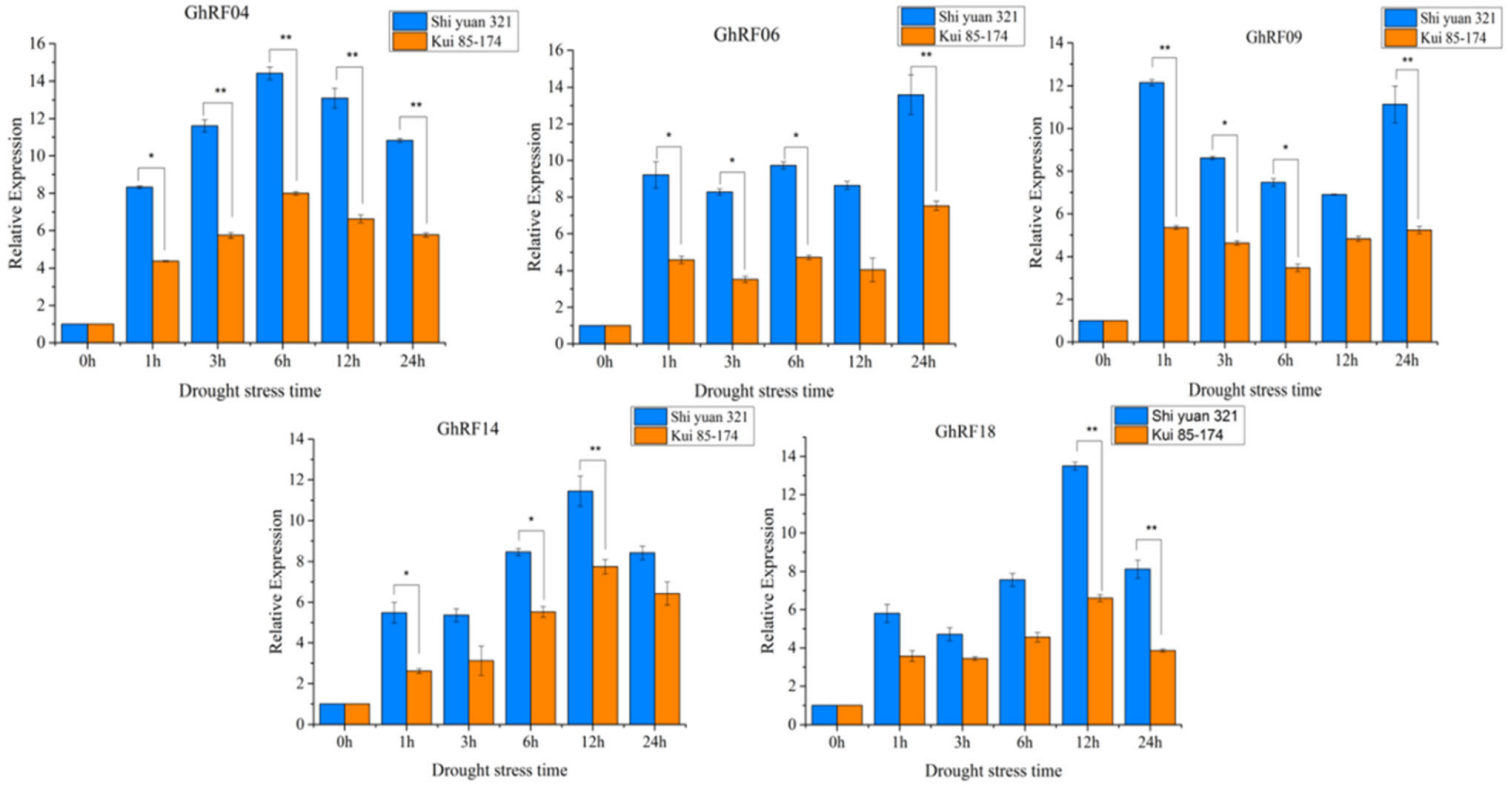
Expression patterns of five *GhRF* genes in Shi yuan 321 and Kui 85–174 under drought stress

Throughout the research process, RNA-seq data analysis of each GhRF gene after drought stress provided an important basis for functional analysis (Fig. 5). According to the qRT-PCR results, five of these genes were significantly upregulated (> fivefold) in Shi yuan 321 and Kui85-174 (Fig. 6). The results are shown in Fig. 6. These genes are involved in the response to drought stress. An analysis of variance of the RF genes between Shi yuan 321 and Kui 85–174 was performed, and the results showed that the RF gene may be involved in the drought stress response (Fig. 6). The differential expression analysis of these genes showed that cotton RF genes may regulate complex cellular processes to affect different physiological processes and improve tolerance to drought stress. For example, *GhRF04*, *GhRF06*, *GhRF09*, *GhRF14* and *GhRF18* were induced after drought stress, showed high expression, and contributed to alleviating drought stress (Fig. 6).

## Conclusions

In this study, we identified 25 members of the RF gene family in upland cotton. Through phylogenetic tree analysis, these genes were divided into three main groups. qRT-PCR analysis showed that the RF gene family exhibits characteristic expression patterns induced by drought stress. Some of the RF genes may be involved in the drought stress response, such as *GhRF04*, *GhRF06*, *GhRF09*, *GhRF14*, and *GhRF18*, and their expression is significantly induced by drought treatment. The expression levels of these five RF genes were significantly higher in drought-resistant variety (Shi yuan 321) than in drought-sensitive variety (Kui85-174). Therefore, our analysis of the cotton RF gene family increases our understanding of the role of RF genes in drought stress, and helps us to further understand the function of the *GhRF* family genes. This increased understanding provides a theoretical basis for the genetic improvement of drought tolerance in cotton.

## Supplementary Information

Below is the link to the electronic supplementary material.

Supplementary file1 (XLSX 12 kb)

Supplementary file2 (XLSX 11 kb)
